# Bibliographical Notices

**Published:** 1856-08

**Authors:** 


### BIBLIOGRAPHICAL NOTICES.

*Obstetrical Memoirs and Contributions.* By James Y. Sympson, M. D., F. R. S. E., &c., &c. Edited by Drs. Priestley of Edinburg, and Dr. Storer of Boston. J. P. Lippincott & Co., Phila., 1856.

This work is a collection of papers, written by the distinguished author at different times, and published through different channels of medical literature. The articles are various, and many of them upon subjects not often treated, and full of very curious information. Many of the subjects are of the highest practical importance, and treated with the usual ability of the writer, who is so well informed, and so generally experienced in all that belongs to obstetricy. It is not, as will be inferred, a systematic work on Obstetrics, but treats of subjects arranged under the following heads: "Pathology of the Puerperal State," "Physiology and Pathology of the Products of Conception," "Pathology of Infancy and Childhood, and Anæsthesia."

Under the first three heads, many subjects are new to the profession generally, and are highly suggestive and instructive. The subject of the last head, seems but an enlargement of a volume by the same author, heretofore published in this country. Prof. Sympson has, perhaps, done more to encourage the use of anæsthetics, than any other man, and if such results as he claims were confidently expected by the profession generally, their use would soon become universal.

We do not propose to discuss the merits of this agent, but simply to state that they have not met with such general acceptance in this country as the learned Professor would teach they deserved, and (we say it with great deference) we fully concur with our countrymen, in the more prudential use of such powerful agents, notwithstanding the high authority with which we might defend their more indiscriminate application.

The volume, upon the whole, however, is of great value, and we can safely recommend it as deserving perusal. The enterprising publishers have executed their part in beautiful style.

We received this work through the hands of our worthy friend, R. B. Ogden, Bookseller, of this city.

*Wood's Therapeutics and Pharmalcology.* Lippincott & Co., Philadelphia.

We have this moment received the above work, and, of course, as the Journal is about going to press, we are not prepared to give a lengthy notice of it.

The work is introduced to the attention of the reader, by a preface in which he explains its design, and the circumstances requisite to a correct appreciation of it.

In chapter 1st, he discusses the operation of medicines, which he divides into *primary* and *secondary.*

The first division embraces their operations through the circulation, or by absorption—through the nerves, their local primary operation, and the modes of their primary operation—mechanical, chemical, physiological, vital or dynamic.

The secondary operation of medicines he divides into heads, and discusses each in turn as follows: 1. Depression after excitement. 2. By reaction after depression. 3. Through dependence of function. 4. Through sympathy or nervous transmission. 5. Through revulsion or derivation. 6. Through the repair of injuries. 7. Through the removal of the cause.

The Second Chapter. 1. Effects of medicines, estimation of their powers or effects, which last he again subdivides and severally treats as "Through their sensible properties—their chemical relations—Botanical affinities"—experiments therewith upon the lower animals, and observations—effects on man.

2. Whether their effects are organic or functional.

3. Characteristic effects of medicines.

4. Influences modifying the effects of medicines, as sex—temperament—idiosyncrasy—disease—climate—habit—modes of living—mental action.

The Third Chapter. Treats of the application of medicines.

The Fourth Chapter. The classification of medicines.

We have only given a short abstract of the subjects treated of in the first volume, and we regard this indeed as a work of supererogation, when we consider the high reputation which the author has already acquired. His work on Practice, and his share of the labors of the Dyspensatory, have given him a world wide reputation, and, therefore, any remarks which we may make, will not add to that which is conceded by all.

This work also came through the hands of our obliging friend Ogden, of this place, with whom arrangements can be made for procuring the work by applying soon—and we hope it will prove a familiar member of the library of each of the profession of the State. The execution of it by the publishers is such as to convey their high appreciation of it.

*The Cause and Curative Treatment of Sterility, &c.* By Augustus K. Gardiner, A. M., M. D., &c., &c.. Dewitt & Davenport, New York, 1856.

We have examined, with such care as our time allowed, this beautiful work. The publishers have gotten it up in fine style, the binding neat, the wood cuts well executed, and the colored plates beautifully executed. The work is pretty thorough in its way generally, but not as full on some points, as we should like to see a monograph devoted to this interesting subject.

Generally the doctrines laid down are such as are now adopted by the profession; but as there are many points of interest which have not as yet been fully demonstrated, and which from this very intricacy must require farther and very minute enquiry, we think there will be fouud many who cannot give their unqualified assent to all of them. We know it has long been the theory that the period of menstruation in the human female is identical with the period of *heat* in some of the lower mammalia. We cannot however, see the identity or even the physiological analogy. Dr. Gardiner gives in his adherence to this theory. Prof. Dunglison and many other able physiologists deny the alledged analogy. The human female is too far above the other mammiferous animals in her organization and her various functions, to be likened to them. But we cannot pursue this subject. Before quitting this point of the subject, however, we wish to say a few words about the statement made to Dr. G. on the authority of a medical army officer that" The Indians of Puget Sound have a season of amorousness affecting both sexes —from May to October, most active in June, corresponding with the salmon-eating season." Is is not probable that the above-mentioned amorousness is attributable to the aphrodical properties of the salmon? We will only mention one more statement made by Dr. G. to which we think objection may be made. On pages 56 and 59 he says, "Within three or four weeks of the first division of the germ vesicle the foundations of the eyes, limbs and the spinal cord are laid. In two more weeks traces of forming bone are observed, a face is sketched out and fingers may be traced. In another two, a forehead, lungs and ribs have been added. In another month, a rudimentary brain and a two-sided heart. In two months additional the nails and teeth bud, and in four months beyond this every organ in the scheme that is necessary for the accomplishment of human life. Then this growth of developement ceases, thenceforward all growth is that of augmentation."

We doubt very much whether all after growth is simply augmentation. The permanent teeth, the nervous system, especially the brain, the generative organs, all undergo a change different, in our opinion, to simple augmentation. In fact other portions of the system are not fully developed for a long time after birth.

Dr. Gardner has given due credit to the views and labors of American Physicians, wherever he has been able to do so. To Dr. Sims he dedicates his work, and to his skill pays a well merited tribute. To Dr. Meigs he refers for the purpose of elucidating his subject, and we are pleased to know that, by him, that able, eloquent and accomplished teacher, is fully appreciated .Dr. Meigs has met much opposition from American reviewers, much abuse, and been reviled where he should have been praised. He has done much for the profession, and it is a pleasure to give him honor therefor, though he needs no praise from us.

We again recommend the work of Dr. Gardner to the consideration of the profession.

*Human Physiology, Statistical and Dynamical.* By John William Draper, M. D., L. L. D., Prof. Chemistry and Physiology in the New York University. Harper & Brothers, New York, 1856.

We have upon our table the above work, and have had time to give it but a general glance. So far as we can judge from such an examination, the work is of decided merit. It is a well condensed view of the present state of the science, stripped of much of its technicality. The author desiring to reduce the mode of investigating the subject more towards the inductive method, now so well applied to the investigation of the more exact sciences, has not been altogether fruitless, and we hope will have his just influence in the farther developments of this important and interesting subject. It will also be seen that he presents, in an attractive manner, the relations borne by physiology to moral and religious condition; an aspect, though not hitherto regarded as strictly professional, is every way worthy the attention of those to whose direction are often committed the care of the physical man. As a profession, we are charged with materialism and infidelity, but Prof. Draper shows in what materialism consists, and how far its tendencies are to infidelity, without directly discussing the subject, or attempting to repel the imputation. None will be the worse christian from reading him.

With these peculiarities, the work may be recommended as a concise, well arranged, and beautifully illustrated presentation of physiology as it is, and commend it to the profession as such.

The distinguished publishers, as usual, have done every thing necessary in their department, and have our thanks for the copy presented us.

Died.—At his residence in Nevada, Iowa, May 6th, 1856, of stricture of the bowels, N. A. Kellogg, M. D., æt. 29 years.

In the death of Dr. K., the profession, as well as the citizens of Story County, have suffered a loss that will, with difficulty, be repaired. Dr. C. was an alumni of the Medical College of Ohio.

Though young in years, he left a family and a large circle of friends to mourn his untimely loss.

